# Formation of Polarized, Functional Artificial Cells from Compartmentalized Droplet Networks and Nanomaterials, Using One‐Step, Dual‐Material 3D‐Printed Microfluidics

**DOI:** 10.1002/advs.201901719

**Published:** 2019-10-24

**Authors:** Jin Li, Divesh Kamal Baxani, William David Jamieson, Wen Xu, Victoria Garcia Rocha, David Anthony Barrow, Oliver Kieran Castell

**Affiliations:** ^1^ Cardiff University School of Pharmacy and Pharmaceutical Sciences Redwood Building, King Edward VII Ave Cardiff CF10 3NB UK; ^2^ Cardiff University School of Engineering Queen's Buildings, 14‐17 The Parade Cardiff CF24 3AA UK; ^3^ Cardiff Business School Cardiff University Aberconway Building, Colum Dr Cardiff CF10 3EU UK

**Keywords:** 3D‐printed microfluidics, anisotropic materials, artificial cells, biomimetic materials, compartmentalization, droplet interface bilayers, group behavior, lipid bilayers

## Abstract

The bottom‐up construction of synthetic cells with user‐defined chemical organization holds considerable promise in the creation of bioinspired materials. Complex emulsions, droplet networks, and nested vesicles all represent platforms for the engineering of segregated chemistries with controlled communication, analogous to biological cells. Microfluidic manufacture of such droplet‐based materials typically results in radial or axisymmetric structures. In contrast, biological cells frequently display chemical polarity or gradients, which enable the determination of directionality, and inform higher‐order interactions. Here, a dual‐material, 3D‐printing methodology to produce microfluidic architectures that enable the construction of functional, asymmetric, hierarchical, emulsion‐based artificial cellular chassis is developed. These materials incorporate droplet networks, lipid membranes, and nanoparticle components. Microfluidic 3D‐channel arrangements enable symmetry‐breaking and the spatial patterning of droplet hierarchies. This approach can produce internal gradients and hemispherically patterned, multilayered shells alongside chemical compartmentalization. Such organization enables incorporation of organic and inorganic components, including lipid bilayers, within the same entity. In this way, functional polarization, that imparts individual and collective directionality on the resulting artificial cells, is demonstrated. This approach enables exploitation of polarity and asymmetry, in conjunction with compartmentalized and networked chemistry, in single and higher‐order organized structures, thereby increasing the palette of functionality in artificial cellular materials.

## Graphical abstract

Polarized artificial cells are created by 3D‐microfluidics. Multi‐material 3D‐printed microfluidics is used to create hierarchical droplet assemblies, where 3D‐channel geometries enable asymmetric and polarized patterning in droplet assemblies. These materials contain lipid‐bilayer‐segregated droplet networks and integrate patterned organic and inorganic components. Polarized patterning with magnetic microparticles bestows directional properties and motion in aqueous environments, with constructs exhibiting simple group‐level behaviors.

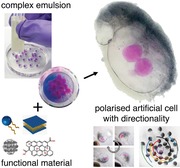

## 1 Introduction

The creation of artificial cells with functional properties, that are analogous to their biological counterparts, is envisaged to give rise to a wealth of opportunities, in a diversity of application areas, in both biotechnology and therapeutics,^1^, ^2^, ^3^, ^4^, ^5^ for example, in tumor cell targeted drug delivery^6^ and artificial kidneys for clinical treatments.^7^ Imparting functionality to artificial cells is typically underpinned by organization of chemical reactions, with the aim to engineer specific dynamic or responsive behaviors. This itself is enabled by the spatiotemporal organization of chemical reactions, and their selective, controlled communication.^8^, ^9^, ^10^, ^11^, ^12^ The aspiration to engineer such chemical models which harness biology, represents a conceptual shift from linear systems with fixed physical‐chemical properties, toward the exploitation of emergent, nonequilibrium, and dynamic properties.^13^ Such approaches are both inspired by, and become increasingly realizable with, our understanding of biological complexity, and the cell's exploitation of emergent behaviors; for instance, those arising from (i) molecular self‐assembly, (ii) the barrier properties of membranes, (iii) the compartmentalization of multiple chemistries, and (iv) the process of symmetry‐breaking, to develop new properties.^14^, ^15^, ^16^, ^17^, ^18^, ^19^, ^20^, ^21^, ^22^ In this regard, and of particular interest, is the ability to manufacture miniaturized, asymmetric entities with spatially organized chemistries to dictate functional polarity.^23^ Such features have been demonstrated to possess emergent and collective properties in solid‐state materials.^24^, ^25^ In biological systems, these properties are essential for dictating directional motility,^26^ governing cellular migration,^27^ enabling response to external chemical gradients^28^ and defining directional growth.^29^, ^30^ Consequently, there has been significant interest in incorporating such polarizable properties in artificial cells.^31^, ^32^, ^33^, ^34^, ^35^, ^36^, ^37^, ^38^ However, to date, the practical establishment of this has remained limited.^39^, ^40^

Despite the importance of discrete chemical organization in artificial cells, the majority of research in bottom‐up synthetic biology, has focused on the development of much simpler models. There has been significant interest in the construction of spontaneous, self‐assembled, artificial cells, in the form of vesicles, colloidosomes, or phase‐separated oil–water, or water–water (coacervate) systems.^41^, ^42^, ^43^, ^44^, ^45^ The exploitation of such processes can shed important light on the emergence of early life, and resultant structures may represent minimally complex, artificial cell chassis, often termed “protocells.” However, using these spontaneous self‐assembly processes alone, it is challenging to recapitulate the controlled subcompartmentalization and spatial organization of biological cells, and, therefore, the higher functionality of even simple biological systems. In this regard, it is notable that few biological structures are formed de novo, instead being spatially and temporally constructed, or formed, from existing structures.^46^ Consequently, droplet microfluidics represents an interesting parallel, where channel architecture and flow provide a route for directing the assembly of compartmentalized, and spatially patterned, chemically organized, soft‐matter materials.^22^, ^47^, ^48^, ^49^

Beyond these self‐assembled systems, increased structural complexity has been sought in droplet networks^50^, ^51^, ^52^ and compartmentalized vesicles^53^ to produce interconnected, membrane‐segregated compartments, as artificial cellular systems. Such materials have been constructed manually,^18^ by the printing or assembly of individual droplet units^20^ and by using microfluidics^54^ and demonstrated as networked reactors possessing emergent properties.^55^, ^56^ Microfluidic construction of such materials offers the opportunity of scalable production, using massively parallel microfluidics,^57^, ^58^ and typically involves their formation from hierarchical water/oil emulsions. Ourselves, and others, have demonstrated the creation of droplet networks, segregated by lipid bilayers using microfluidic approaches.^54^, ^59^ By extending this concept to a third phase and forming a triple emulsion, such droplet structures can be effectively encapsulated within a permeable, freestanding, hydrogel shell. This droplet assembly is able to communicate with its external environment and be functionally enhanced by the integration of transmembrane proteins.^59^

It is notable that the microfluidic formation of such hierarchical droplets, or higher‐order emulsions, requires the integration of both hydrophilic and hydrophobic surfaces within the same microfluidic device. This determines the channel wetting properties and governs whether water or oil droplets form at a given junction. Consequently, the requirement to alternate between aqueous and nonaqueous wetting phases represents a fabrication challenge to achieve the integration of both hydrophilic and hydrophobic channels. This may entail the complex assembly of multimaterial, or surface‐functionalized, fluidic channels. Furthermore, traditional planar fabrication tools usually constrain droplet flows to a single plane, which creates 2D axisymmetric morphologies in double and triple emulsions. Consequently, in both lipid bilayer stabilized, and more traditional multiphase emulsions, a number of constraints are placed on the ability to create nonsymmetric droplet structures in a well‐controlled manner.

In this regard, asymmetric droplets, or so‐called Janus droplets, have been explored as a means to break the morphological symmetry in droplets.^60^, ^61^, ^62^ Janus droplets were first reported by Nisisako et al with microfluidic formation of such structures deterministically created in single emulsion laminar flows.^63^ Janus particles have been used in applications that exploit the particle anisotropy, ranging from chemical sensors^64^ to electrically activated, reconfigurable displays.^63^ Double emulsions have also been used as a template to create Janus polymer structures, with the immiscible oil and water volumes templating the synthesis of two conjoined particle hemispheres.^65^ Double emulsions with a Janus shell have also been reported,^66^, ^67^ but the Janus patterning has remained limited to a single phase, and in a single plane. The ability to expand such approaches and combine with droplet interface bilayer networks could provide a means to pattern individual layers of hierarchical, membrane‐based, cellular structures. Further, the ability to create droplet structures with symmetry‐breaking in more than one plane would enable the discrete patterning of each phase, and its orientation, in the droplet hierarchy. As a result, this could produce chemical gradients or polarized phases, and position compartments spatially within the fluidic or soft matrices. This would, therefore, facilitate the construction of synthetic cell chassis, with compartmentalized chemistries, access to membrane biochemistry, with overall spatial control of assembly. Such order would enable control over chemical polarity, and increase the functional complexity of artificial cells.

To realize this, we report here the development of one‐step, multimaterial microfluidic devices with 3D arranged channels, to produce hierarchical droplets with discrete patterning of component liquid phases. Using this approach, we demonstrate the production of symmetry‐breaking droplet structures, incorporating patterned organic and inorganic components, with biological lipid bilayers for the first time. This increases the palette of functionality and afforded assembly control in such chemically organized synthetic cells. We demonstrate the ability to impart polarization‐driven behaviors in the constructs, by creating magnetically polarized, encapsulated droplet networks that can orientate, rotate, and migrate, as well as collectively establish a common orientation, and undergo shared collective motions in larger populations.

## 2 Results

### 2.1 Single‐Step 3D‐Printed Microfluidic Devices for the Production of Multiphase Emulsions

**Figure 1:**
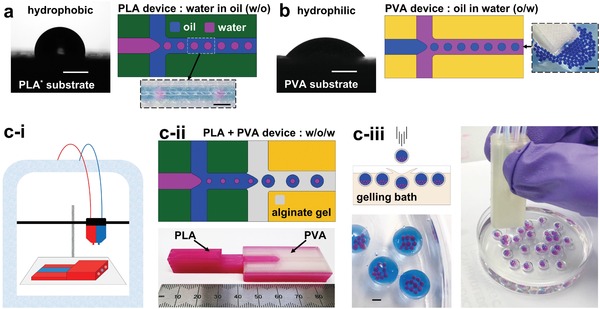
3D‐printing of microfluidic devices in hydrophobic and hydrophilic polymers enables the production of both water droplets in oil and oil droplets in water. Dual‐material printed microfluidic devices allow the sequential application of these droplet formation regimes to produce triple emulsions of hierarchical water/oil/water droplet structures. a) 3D‐printed polylactic acid (PLA) substrate displays hydrophobic properties demonstrated by water contact angle measurement. 3D‐printed PLA T‐junction generates water droplets in oil (schematic and experimental image). b) 3D‐printed polyvinyl alcohol (PVA) substrate demonstrates more hydrophilic properties by water contact angle measurement. 3D‐printed PVA T‐junction generates oil droplets in water (schematic and experimental image of oil (blue) droplet ejection from PVA device into a water bath). c‐i) Dual head extrusion printer enables fabrication of integrated PLA and PVA, dual material, microfluidic devices. c‐ii) (Top) Schematic depiction of operational concept; sequential, droplet generating, flow‐focusing junctions in PLA and PVA, enable the formation of double emulsions (water‐in‐oil‐in‐hydrogel (ungelled) (w/o/w)). Monolithic device realized in lower image, comprised of PLA (pink) and PVA (colorless) materials. c‐iii) Triple emulsions formed as hydrogel droplets are ejected from the microfluidic device. The outer, liquid, alginate phase is gelled upon dripping into a CaCl_2_ containing gelling bath, creating a triple water‐oil‐water emulsion with a gelled outermost shell. These complex emulsions (pink: aqueous internal phase, blue: oil midphase, colorless: outer alginate shell phase) are individually deposited from a dual material, microfluidic device. All scale bars: 1 mm.

The advent of 3D printing has enabled the construction of 3D fluidic architectures.^68^, ^69^ In droplet microfluidics, hydrophobic fluidic channels govern the formation of water droplets in a continuous oil flow, and hydrophilic fluidic channels enable the dispersion of oil droplets in continuous water flows. Here, we developed a monolithically constructed, 3D fluidic device, consisting of both hydrophobic and hydrophilic materials, alternately deposited in a single, integrated build process, using a dual‐material printer (Ultimaker 3). This created both hydrophilic and hydrophobic ducts in a single fabrication step, without the requirement for surface modification, or post‐manufacture integration of different substrates. Using this approach, it was possible to produce water droplets in oil using a hydrophobic PLA channel (**Figure** **1**a), and oil droplets in water, within a more hydrophilic polyvinyl alcohol (PVA) channel (Figure 1b). In Figure 1a,b, the aqueous wetting contact angle of PLA and PVA is depicted, together with the resultant droplet regimes. The increased hydrophilic properties of PVA enable inversion of the usual droplet formation regime to produce oil droplets in water. These material architectures were then combined in a single device to sequentially produce water droplets in oil, followed by oil droplets in water, and finally, the ejection of water droplets from the device, to a collection bath (Figure 1c, and Table S1, Video S1, Supporting Information). This enabled the hierarchal assembly of complex, multiphase emulsions, using the monolithic microfluidic device, which was fabricated in a one‐step process. Figure 1c‐iii demonstrates the production of aqueous droplets (red) encapsulated within an oil droplet (blue), itself encapsulated within an alginate hydrogel shell (colorless) using this approach. These triple emulsions are shown to be sequentially deposited from the fluidic device, in effect, “printing” individual complex emulsion droplets.

### 2.2 Characterization of Complex Emulsion Formation

**Figure 2:**
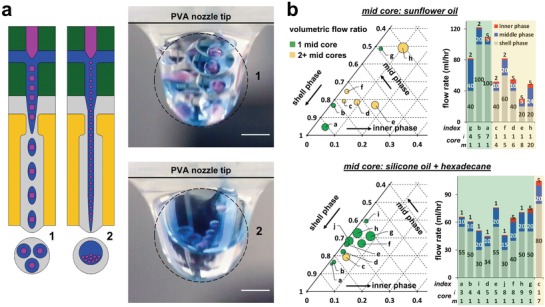
Tuning fluid flows enables control of droplet morphology. a) The PVA junction responsible for oil‐in‐water droplet formation is capable of operating in two distinct droplet generating regimes in the formation of triple emulsions; either by dripping (1: left and top) or jetting (2: right and lower). These result in the formation of multiple (1: left and top) or single (2: right and lower), encapsulated middle phase oil droplets (blue), containing inner phase aqueous droplets (pink) within the larger emulsion construct (gray). Images (right) depict tipple emulsion formation at the device exit, under dripping (top) and jetting (lower) regimes. b) Ternary phase diagrams depicting flow ratio, and stacked column graphs detailing volumetric flow rates, of the three input phases and corresponding triple emulsion morphology. These detail the number of middle‐phase droplets (*m*) and their number of constituent internal aqueous droplets (*i*), for operation with two different oil phases. On each plot, yellow illustrates two or more midphase oil droplets as a result of operation in a dripping regime, and green indicates a single midphase oil droplet from operation in a jetting regime. Index labels (a–j) enable cross‐correlation between plots. Marker size on phase diagram denotes number of encapsulated inner aqueous droplets (*i*), with value quoted under stacked column graph (*n* = 30 under each condition).

The formation of oil droplets in a continuous water phase, without surface modification, is usually challenging, owing to the native preferential hydrophobic wetting of many substrate materials, which favor the formation of water droplets in oil. Consequently, to date, only water droplet formation, in extrusion 3D‐printed microfluidic devices, has been demonstrated.^70^ We observe that in the formation of complex emulsions, the comparatively hydrophilic PVA junction is capable of operating in two distinct droplet generating regimes for the formation of oil droplets in water. These regimes are essentially, either dripping or jetting (**Figure** **2**a), both being determined by the combination of input fluid flow rates, and the specific composition of the respective fluid phases (Figure 2b and Table S2, Supporting Information). In the formation of triple emulsions, this results in the formation of either single, or multiple, encapsulated middle phase oil droplets (designated *m*), containing inner phase aqueous droplets (designated *i*) in the larger emulsion constructs, under jetting and dripping regimes, respectively. Figure 2b shows the manifestation of this with respect to the relative, and absolute, flow rates of the three input fluid phases, for both a fatty‐acid‐rich sunflower oil, and a hexadecane/silicone oil midphase. The complex interplay of fluid flow properties has been characterized in the determination of droplet formation, by dripping and jetting regimes and their transitions.^71^ Fluid density, velocity, droplet and channel diameter, surface tension and viscosity, all play an important role, as drag and inertial forces compete with surface tension.^72^, ^73^ It is unsurprising that this relationship becomes more complex in triple‐phase systems, where flow rates govern both upstream droplet formation, and also influence linear flow velocity downstream at subsequent encapsulation. The fluid dynamics of droplet formation are additionally perturbed by both internal and external immiscible interfaces.^74^ New dimensionless numbers have been proposed to describe triple emulsion droplet formation,^75^ albeit by a different microfluidic method with fluid phases unsuited to the creation of artificial cells. In this work, we take an empirical approach to control the number of inner (*i*) and middle droplets (*m*) that are encapsulated in the shell phase, by the tuning of fluid flow rates, to govern the droplet generation frequency of each of the three phases. The output triple emulsion droplets have uniform morphology (Video S1, Supporting Information), and relatively monodisperse size distributions (<4%) for each phase (Figure S1, Supporting Information). The size of the internal and midphase droplets is observed to change with the relative flow rates of the component fluid phases, corresponding to the internal and midphase droplet number. This is further detailed in Figure S2 (Supporting Information).

### 2.3 3D Microfluidic Architectures Enable Formation of Asymmetric and Patterned Complex Emulsions, Creating Hierarchal Polarized Structures

**Figure 3:**
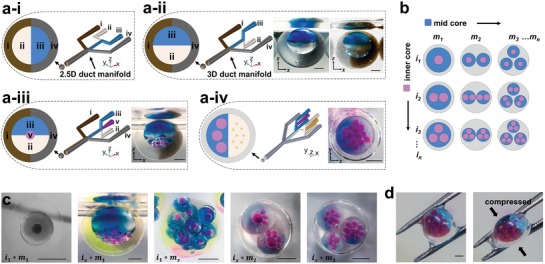
3D configurations of dual‐material fluidic channels enable the spatial patterning of complex emulsions. a) (i–iv) Four different fluidic channel geometries. The ability to manufacture channels and junctions in 3D space (ii–iv) provides new opportunities for the spatial patterning and organization of multiphase emulsions, compared to traditional planar (2.5D) channel arrangements (a‐i). a‐i) With fluidic channels constrained in a single plane, Janus‐core and Janus‐shell (bi‐Janus) double emulsions can be formed, with the hemispherical divide occupying the same orientation, in both instances. a‐ii) Rotating the plane of one droplet generating flow focusing junction geometry by 90° results in the rotation of the plane of Janus droplet formation. Here, creating the depicted bi‐Janus droplet hierarchies, with independent and opposing Janus orientation (90° rotation) in the core (mineral oil with and without oil blue N) and shell phases (alginate shell with and without silica particles or graphene oxide). a‐iii) The addition of a fifth aqueous input (*v*) upstream of the consecutive hydrophobic and hydrophilic droplet generating geometries of (a‐ii), enables the addition of internal aqueous droplets (pink) as inner droplets in bi‐Janus triple emulsions with perpendicular Janus patterning. a‐iv) Extension of this principle to two independent upstream aqueous droplet generating geometries, combined to produce a parallel coflow, enables the addition of two types (differing size and contents) of aqueous internal droplets within the resultant triple emulsion. The lateral offset delivery of these droplets into the common channel, defines the position of entry in the encapsulating droplet. Small aqueous droplets (yellow) are retained to the right‐hand perimeter of the larger (pink) aqueous droplets within the Janus oil middle phase of the triple emulsion. Complementary CFD modeling of the emulsion stabilization within fluidic duct can be found in Table S4 (Supporting Information). b) Schematic illustration of parameter space, droplet number, and arrangement in triple emulsions (see also Figure 2). c) In combination, the spatial patterning enabled by 3D fluidic geometries demonstrated in (a) with the ability to control number of inner and middle phase droplets (b), a diverse range of complex and patterned triple emulsions can be made. (*i* and *m* subscripts illustrate droplet numbers of construct). d) The encapsulating hydrogel shell provides mechanical stability, thereby enabling physical manipulation of resultant emulsion droplets. Scale bars: 1 mm.

The use of 3D printing enables the creation of fully 3D microfluidic architectures, unlike more traditional fabrication approaches, which are usually constrained to machining in a single plane. Using the extra‐dimensionality afforded by 3D printing allowed us to create devices which control the phase orientations and encapsulations within the complex emulsions system, thereby creating patterned and asymmetric droplet structures around different geometric axes (**Figure** **3**). The dominance of low Reynolds number flows maintains the laminar streams of fluids, thereby minimizing mixing. Axial symmetry at the droplet generating junctions was used to enable asymmetric fluid additions with respect to the major channel axis. While also creating a symmetric shear force on the disperse (droplet) phase. This maintained the patterning or organization of emulsions, when the dispersed phase was pinched off by the continuous (shell) phase. Complementary computational fluid dynamic modeling finds that the organization within the resultant w/o/w emulsion flow is stabilized in the extended fluidic duct in our experimental conditions (Table S3, Video S2, Supporting Information). We found that, while in flow, the inner aqueous droplets reached their equilibrium positions within the middle oil droplet, without ejection into the continuous hydrogel phase. This process is dominated by the viscous stress, competing with interfacial tension^76^ (see Discussion, Supporting Information). Similarly, we find internal (*i*) droplets are constrained within lateral domains of the encapsulating oil droplet, as are differential additions of middle phase (*m*) oils, thus enabling the maintenance of internal hemispherical patterning and gradients (see Table S4, Supporting Information and Discussion, Supporting Information).

Using this experimental approach, we orientated sequential droplet generating geometries in 3D. This allowed us to spatially pattern the droplet structures with control over the designated patterned phase or phases, and the geometric orientation of patterning. Consequentially, this provided the ability to create both Janus shells, Janus and gradient mid‐droplets (*m*), and achieve the controlled hemispherical placement of inner droplets (*i*). These may differ in size and contents, and be located in different regions within the encapsulating droplet (Figure 3). Figure 3a‐i illustrates conceptually how traditional 2.5D channel architectures can be used to create Janus shells with inner droplets as double emulsions. Enabled by 3D fabrication, it becomes possible to rotate the fluid inlets (by 90° in this instance) providing the opportunity to create asymmetric coflows in different orientations with respect to the traditional single fabrication axis. In this way, it becomes possible to combine multiple coflow orientations, creating both Janus shells and Janus interiors, with independent and opposing orientations in both double (Figure 3a‐ii) and triple (Figure 3a‐iii) emulsions. In principle, this process can be extended to increasing hierarchies of droplet structures in complex emulsions.

We also demonstrate that this approach can be extended to the production of differing inner droplets (*i*) in the triple emulsion, and dictate their location in the final droplet construct. This is achieved by joining two streams of water/oil emulsions at a Y‐shaped junction to form a Janus flow before its breakup into discrete encapsulated droplets (Tables S1 row 3 and S4, Supporting Information). Figure 3a‐iv illustrates the production of inner droplets (*i*) of two different sizes and contents (red ≈430 µm and yellow ≈125 µm) within a Janus encapsulating oil droplet (*m*), itself within a hydrogel shell. The smaller (yellow) inner droplets are retained to the right‐hand perimeter of the larger red droplet population. With this approach to spatial patterning, enabled by 3D fabrication, in combination with the flow‐rate controlling size and number of inner and midphase droplets (Figure 3b), we demonstrate that it is possible to produce a complex diversity of spatially compartmentalized and patterned, triple emulsion structures (Figure 3c) from these new 3D‐printed, microfluidic devices. The outer shell phase is gelled on exit from the microfluidic device by the Ca^2+^ quickly diffusing and crosslinking the alginate shell in the gelling bath. As such, the final droplet structures are freestanding and able to withstand mechanical manipulation (Figure 3d). The alginate shell remains water permeable, thus enabling communicative access from the environment to the internal contents, where inner droplets can be segregated from the hydrogel shell by a biomimetic, lipid bilayer.^59^

### 2.4 Functionalized Encapsulated Droplet Interface Bilayers (eDIBs)

In an immiscible, aqueous–oil system, phospholipids may serve as an effective amphiphilic surfactant, stabilizing aqueous droplets in oil through the self‐assembly of lipid monolayers at the water–oil interface. The contacting of two such interfaces leads to the spontaneous creation of a lipid bilayer, resembling the foundational structure of the cell membrane and membrane‐bound organelles.^50^, ^59^ In this way, membrane compartmentalized systems can be constructed within hierarchal droplet architectures, where functional membrane proteins can be reconstituted to enable both the communication and transfer between different compartments and between compartments and the environment.^59^

**Figure 4:**
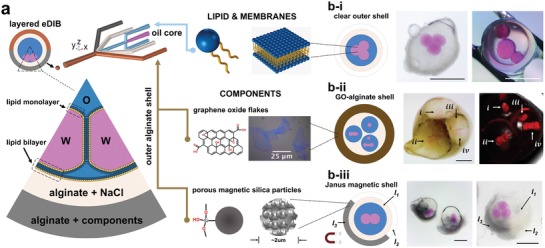
In the presence of phospholipids, droplet networks segregated by lipid bilayers may be formed within a triple emulsion. This creates encapsulated droplet interface bilayer networks (eDIBs) that can be patterned with functional materials using the fluidic patterning approach illustrated in Figure 3. a) The presence of amphiphilic phospholipids in the oil midphase serves as a surfactant, forming self‐assembled lipid monolayers, at each oil–water interface. b‐i) The contacting of two such aqueous interfaces results in the formation of a lipid bilayer between the aqueous volumes, creating lipid membranes between internal aqueous droplets, and the internal droplets and the hydrogel shell, where they make contact. Incorporation of two successive outer alginate coflows, in emulsion formation, can be used to form double‐layered hydrogel shells. These can be used to encapsulate droplet networks with Janus‐, or whole‐shell‐, patterning that incorporates functional materials, such as graphene oxide or silica magnetic particles. b‐ii) Bright‐field and fluorescent images of encapsulated droplet interface bilayers (eDIBs), with an outer hydrogel shell loaded with atomically thin graphene oxide (GO) sheets. b‐iii) Encapsulated droplet interface bilayers (eDIBs) with a double hydrogel shell, comprising an inert spacer shell, and an asymmetric Janus‐shell. The Janus shell incorporates silica magnetic microparticles to impart polarized magnetic properties on the membrane‐based artificial cell construct.

Expansion of the microfluidic patterning approach described earlier was implemented for the creation of encapsulated, droplet interface bilayer systems with layered hydrogel shells. Within these constructs functional organic and inorganic materials were incorporated alongside lipid bilayers (**Figure** **4**a and Video S3, Supporting Information). In this way, we produce eDIBs (Figure 4b‐i), with 25 µm chemically exfoliated graphene oxide (GO) sheets incorporated into the outer shell at concentrations of up to 1.44% w/w (Figure 4b‐ii) (GO characterization Figure S2, Supporting Information). GO is an atomically thin 2D, functional nanomaterial that has attractive mechanical, electrical, chemical, and photonic properties.^77^ GO has been used to nonspecifically bind proteins and peptides,^78^ thus it may be possible to serve as a protective shell layer to protect lipid bilayers from destabilizing peptides and proteins, while retaining the capacity for water and small molecular diffusion to the lipid bilayer. At higher concentrations, the presence of GO increased the viscosity of the hydrogel phase. While this did not hamper the microfluidic formation of the hierarchical droplet assemblies, the slower surface‐tension‐driven relaxation of the outer droplet to a spherical shape during gelation resulted in the gelation of tear‐drop, or ovoid‐shaped, outer shells.

We also produced eDIBs with a functionally inert inner shell of osmotically matched buffer, followed by a second Janus outer shell, containing embedded, 2 µm porous magnetic silica microparticles. In this way, a thin protective inner hydrogel region could serve as a spacer, in effect, isolating larger silica particles from direct contact with the lipid bilayers, but maintaining aqueous and diffusive continuity of the shell with the membrane. Silica particles have been reported to disrupt artificial bilayers^79^ and we observed results consistent with this in the absence of the additional inner hydrogel layer. By the creation of two‐layered shells, we could produce stable particle‐laden encapsulated bilayer systems, with polarized properties, such as a Janus shell (Figure 4b‐iii), or an asymmetric encapsulated droplet network (Figure S3, Supporting Information). In these systems with inner (*i*) aqueous droplets containing fluorophore sulforhodamine B, fluorescent imaging 3 d following manufacture demonstrated that the lipid bilayer networks remained intact (Figure S4, Supporting Information). The modulation of salt conditions of the alginate phase to avoid excessive osmotic stress on the assembled lipid bilayers was also observed to slow the alginate gelation processes, giving rise to some shape fluctuation in the shell of the final constructs, as they gel on entry to the gelling bath, usually giving rise to slight elongation of the overall structure. It should be possible to expand our reported microfluidic methodology to employ in‐channel gelation,^59^ to facilitate gelation while maintaining sphericity within the in‐channel flow. The methodology reported here provides a route for the fabrication of artificial cell chassis containing both lipid membranes and functional nano‐ and microparticulate materials in spatially organized architectures. By using controlled spatial organization, these materials may, therefore, harness the functionality of both the biological and nonbiological components, while circumventing the physical incompatibilities that would otherwise hamper realization. In this way, designer hierarchical droplet structures could be created to control bio, chemical, and physical properties.

### 2.5 Polarization‐Induced Properties of Artificial Cell Chassis

**Figure 5:**
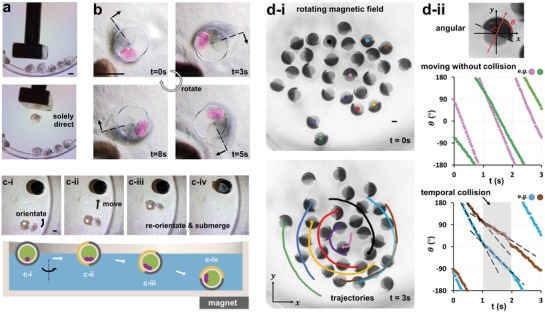
Polarized encapsulated droplet interface bilayers (eDIBs), with an asymmetric Janus‐shell of silica magnetic microparticles, exhibit directional locomotion in an aqueous environment. a) A single magnetically polarized eDIB is corralled from a population with a noncontacting magnetic wand. b) Time sequence of images of a magnetically polarized eDIB, orientating with respect to a rotating magnetic field. The construct rotates clockwise about its central axis. The dotted line indicates the radial boundary of the magnetic and nonmagnetic hemispheres. c) Time‐sequence images of magnetically polarized eDIB orientating and migrating in three dimensions to a magnetic target beneath the Petri dish. c‐i) Orientation in the plane to face the magnet. c‐ii) Migration on the surface plane. c‐iii) Reorientation of the magnetic hemispherical face toward the magnet and submersion of the construct. c‐iv) Localization at lower surface of the Petri dish at magnetic target location. d) A population of core–shell, Janus constructs, containing magnetic microparticles in one hydrogel hemisphere, collectively migrate and spin in a rotating magnetic field. d‐i) Tracked trajectories (subset shown for clarity) highlight clockwise migration about a central point. All constructs adopt a common axial orientation, and also spin clockwise about this axis while migrating. d‐ii) The directionality of each construct is observed by the polarized hemispherical face. Colliding constructs experience a shared reduction in angular rotational velocity, temporarily before moving apart, and restoring velocity (see also Video S4, Supporting Information). Scale bars: 1 mm.

The particle‐containing encapsulated bilayer systems can gain specific functionality from patterned and encapsulated components. Here, we demonstrate the polarization‐induced functional properties of the eDIBs complete with an outer Janus shell of embedded, paramagnetic, silica microparticles. The incorporation of paramagnetic particles enables the manipulation of the eDIBs, by an external magnetic field, enabling mobility and orientational control, in aqueous environments (Video S4, Supporting Information). These artificial cell chassis can be moved individually in an aqueous environment (**Figure** **5**a). In a rotating magnetic field, individual constructs orientate with respect to the magnetic field and to the polarity of their Janus shell, thereby providing a directionality to the droplet structure. These behaviors could be combined in an aqueous pool, where Janus shell eDIBs could seek, orientate, move, and submerge to interface with a static magnetic target placed beneath the Petri dish. The preferred orientation of the construct is governed by the polarized patterning of the eDIB structure (Figure 5c). Such polarization‐induced properties, created by the ability to spatially organize and pattern eDIBs, provide the opportunity to recapitulate biological and biological‐like behaviors. These may include the harnessing of polarization in directional control, polarized chemical organization, or the exploitation of anisotropy, for example, in the self‐organization of higher‐order assemblies or artificial tissues. Figure 5d shows a population of core–shell Janus magnetic architectures in an aqueous environment under a rotating magnetic field. All constructs are observed to spontaneously orientate such that their hemispherical division is in the vertical plane. Individual constructs are observed to migrate clockwise, while simultaneously rotating about their own central axis. This is a consequence of both the overall, and locally polarized, magnetic properties of each construct. Colliding constructs are observed to temporarily comigrate with inhibited rotational velocity, before separation and recovery of rotational motion (Figure 5d‐ii and Table S4, Supporting Information, correlation test). The orientation of individual constructs may all be unified with this type of patterning, and their motion within a larger population may influence others within the group.^25^ Such mobility synchronization results in the emergence of simple, group level behaviors. Thus, these principles may serve to pave the way for the design and programming of more sophisticated, population level behaviors, governed by polarized or directional functionality, in membrane‐based artificial cell communities.

## 3 Discussion

In summary, we have demonstrated the feasibility of a new, simple, one‐step process that uses 3D printing to fabricate monolithic, dual‐material, microfluidic devices, which are able to produce both water‐in‐oil and oil‐in‐water droplet emulsions without the need of surface modification. With this approach to device fabrication, we demonstrate the sequential operation of hydrophobic and hydrophilic droplet generating geometries to create hierarchal droplet structures, where flow rates determine the mode of operation, and, govern the number of encapsulated inner and midphase droplets. By exploiting the ability to completely fabricate manifolds in three dimensions, we are able to generate triple emulsions with 3D‐patterned morphologies. This provides the capability to produce not only Janus shell materials but also hierarchical double‐Janus (shell and midphase) droplets with independent control of the hemispherical orientations. These features can be combined with inner droplet populations of differing size, number, and hemispherical location, alongside the ability to create layered and patterned shells. These droplet templates are able to incorporate lipid bilayers, providing membranes between neighboring aqueous droplets, and between inner droplets and their contact of the midphase‐and‐hydrogel interface. This provides the opportunity to harness lipid membrane, and membrane protein biochemistry, together with biochemical compartmentalization and environmental communication. We are able to combine the presence of lipid membranes with the integration of functional organic and inorganic materials, patterned within the same hydrogel matrix shell. The hydrogel encapsulation provides mechanical rigidity and aqueous compatibility, while microfluidic manufacture provides a potential means to scale manufacture through parallelization of capillary structures.^57^, ^58^ 3D microfluidic architectures constructed from dual hydrophilic and hydrophobic materials enable the control of the landscape of hierarchal droplet construction and provides the ability to pattern the morphologies of the droplet hierarchies and break axial symmetry. This enables the creation of polarized droplet structures, which can be further used to impart additional functionality. This is demonstrated here for the first time, via the construction of hemispherically magnetic, encapsulated droplet interface bilayers, enabling controlled orientation, locomotion, and rotation.

The rapid, dual‐material, fabrication process means that device designs can be rapidly iterated and customized for different desired droplet arrangements. Something not previously possible. This in effect creates the possibility of disposable devices for complex emulsion production, or even use as cartridges for droplet printing. Using 3D channel fabrication techniques, it is conceivable that nonhomogeneous, but spherically centrosymmetric, droplet hierarchies may be produced via a radial array of droplet inlet channels into the pre‐encapsulating flow. Current work is directed toward improved understanding of precise inner‐droplet arrangements to further increase design control. In the reported work, the new use hydrophilic PVA serves to demonstrate feasibility of the dual‐material approach, reporting the creation of complex emulsions using a fused‐filament 3D‐printed device for the first time. However, ultimately, the hydrophilic PVA material is nonoptimum, as it is partially soluble in aqueous media, in this case the hydrogel shell phase, over prolonged periods of use. In the immediate term, this time‐limited use may be offset by the rapidity of device manufacture. While the current commercial palette of printable polymeric materials is limited, it is rapidly growing.^80^ Therefore, the prospect of further hydrophilic polymer substrate materials is promising, as 3D printing enjoys continued growth across ever widening application areas. Similarly, advances in printer resolution can be expected to facilitate the production of narrower channel architectures, thereby enabling the miniaturization of patterned emulsions. With the growing interest in 3D printing of soft materials,^81^ the ability to deposit stable patterned, complex emulsions from a 3D‐printed device, as demonstrated here, may serve as the basis for the 3D printing of large‐scale materials, with complex emulsions or artificial cells as the constituent building blocks. This may find use in bioprinting applications, or in the construction of increasingly complex artificial tissues made from populations of synthetic cells, affording increased material complexity, in comparison to single‐phase droplet printing.^20^ The reported spatial patterning of complex emulsions could be applied with incorporated living cells, enabling the concept of biohybrid materials, which could combine membrane‐bound droplets and cells,^82^ or the integration of cells into hydrogels.^70^, ^83^ The ability to impart polarization or directional preferences in cell‐laden constructs may find use in the self‐organization of 3D tissue engineering and repair systems. The engineering of orientation‐dictated, higher‐order assembly of artificial tissues from component cells holds significant promise. Likewise, polarizable artificial cells may take advantage of directional‐specific behaviors, analogous to phototropic or gravitropic behavior in plants, albeit by alternative mechanisms. The ability to create soft‐matter Janus materials with integrated biological components also paves the way for applied uses similar to their solid‐state counterparts, such as in sensing or displays, but with integrated biochemical capabilities.

Droplets, lipid bilayers, soft‐matter compartmentalized structures and functional nano‐ and micromaterials are all the subject of great interest as building blocks of bioinspired engineered materials. The ability to create spatially organized and patterned droplet structures, comprising all these elements within the same entity, will likely serve to increase the sophistication of artificial cell models, as functional materials combining biological, organic, and inorganic material properties. The ability to impart asymmetric, polarized, or gradient patterning on such hierarchal droplets in any desired orientation, at their formation, through the use of 3D‐printed microfluidics, affords a new level of control in the design and functional utility of these materials. Biology routinely exploits the emergent functionality of the barrier properties of membranes, in combination with chemical gradients and polarization, to impart directional or symmetry‐breaking properties in cells, and enable subsequent individual and collective functionality. This serves as inspiration for the next generation of dynamic, functional, synthetic materials, built using these approaches. The data presented here demonstrate the capability to engineer chemical patterning and impart such properties in aqueous compatible, hydrogel‐encapsulated, membrane‐bound droplet networks. This holds significant promise for the advancement of behaviors and functionalities that more closely mimic the sophisticated functionality of biological cells. These patterned, hierarchal droplet materials are operational in aqueous environments, in which they remain freestanding, and retain diffusive communication between the environment and internal membrane‐bound architectures. This renders them promising candidates for applications across the life sciences, both within and outside the laboratory, such as for responsive drug delivery, self‐repairing and reconfigurable materials, and biochemical computation.

## 4 Experimental Section

*Chemicals and Components*: Alginic acid sodium salt, calcium chloride, sodium chloride, silicone oil AR20, hexadecane, chloroform, Oil Blue N, PVA powder, tween 20, and span 80 were purchased from Sigma‐Aldrich. Sunflower oil (pure) was purchased from Sainsbury's. Hydrophilic, magnetic silica microparticle (SiMAG/MP‐DNA 2.0um) was purchased from Chemicell. 1,2‐Diphytanoyl‐sn‐glycero‐3‐phosphocholine (DPhPC) lipid was purchased from Avanti Polar Lipids. Sulforhodamine B was purchased from Sigma Aldrich. Neodymium magnets were purchased from RS Components. Large flake size of graphene oxide suspension was synthesized using a method previously reported by Rocha et al. elsewhere.^84^ The concentration of GO in the water (1.44 wt%) was calculated by weight difference after freezing and freeze drying (48 h) 4–6 g of suspension. Triple emulsion templates are shown in Table S1 (Supporting Information). The preparations of the precursor for the eDIBs formation were as in a previous work.^59^ Calcium chloride solution (0.5 m) was prepared for the outer alginate matrix gelation. To prepare the microparticle containing alginate solutions, the original particle‐containing solution was diluted in deionized water (1% v/v for SiMAG/MP‐DNA, and up to 1.44 wt% for GO slurry), and the alginate powder was added to prepare a 3 wt% alginate solution using a magnetic stirrer (IKA RCT basic safety control) agitated at 800 rpm and 50 °C for 4 h. The solution was stored at 4 °C.

*Triple Emulsion Construct Materials*: Internal aqueous phase contained 0.5 m NaCl with 100 × 10^−3^ m sulforhodamine B (pink) for observation. The oil midphase for eDIB formation was formulated as hexadecane and silicone oil AR20 (1:2 v/v) with DPhPC 8.33 mg mL^−1^ for droplet network production. The oil phase for non‐bilayer constructs was formulated with either sunflower oil, or mineral oil, with surfactant (see Table S2, Supporting Information). Oil Blue N was added at 0.05 wt% for visualization, to demonstrate liquid Janus cores, visualize internal concentration gradients, and their persistence throughout and beyond the microfluidic production process. Shell phases were formulated with 3% alginate solution, containing additional 0.5 m NaCl (for the eDIB inner alginate shell to osmotically match the internal aqueous phase droplet), or GO or silica nanoparticles (for the eDIB outer alginate shell patterning) as described in the text. The gelling bath contained 0.5 m CaCl_2_ solution.

*Microfluidics*: Microfluidics devices were designed and modeled with COMSOL Multiphysics (the dimensions of tubular channels are in the range of 240–1200 µm diameter), and were printed using fused filament fabrication printers (Ultimaker 3) with PLA filaments and PVA filaments (Ultimaker), using 0.4 mm AA and 0.4 mm BB print‐cores. The print g‐codes were programmed using Cura (version 3.3.1) software with customized settings (Figure S1, Supporting Information). The layer height was controlled at 0.06 mm, and the printing speed was tuned at 100 mm s^−1^ with default printing temperatures (200 °C for PLA filaments and 215 °C for PVA filaments). The printed parts were treated with chloroform vapor within an enclosed metal box for 8 min, and the treated pieces were left in a fume cupboard for 2 h before use.^70^ Precursors were loaded in syringes (5 mL, gas‐tight, SGE Analytical Science), and were delivered to the microfluidic devices at a constant flowrate, through PEEK and PFA interconnects and FEP tubing, using syringe displacement pumps (KD Scientific, model 789200L). Details of the controlled sequential emulsification using multiphase microfluidics are given in a previous work.^22^

*Experiments and Measurements*: General images and videos were taken using a 12MP digital camera with mounted zooming lenses on 3D‐printed stands. Epifluorescence and light microscopy images were captured using a modified Nikon Eclipse Ti‐U inverted microscope and Andor iXon ultra 897 EMCCD camera. For white light images, illumination was provided by the microscopes integrated 100 W halogen lamp, while a Shanghai Dream Lasers 532 nm DPSS laser with a power output of 100 mW was utilized for epifluorescence illumination. Laser coupling into the microscope was achieved via a custom‐built optical circuit utilizing components sourced from Thorlabs Chroma and Semrock followed by a single mode fiber‐optic launch. A low magnification 1× (Nikon Plan UW) objective was used in all acquisitions. Excitation and emission fluorescence wavelengths were separated using a 532 nm edge dichroic mirror combined with a 542 nm edge long‐pass filter and a 565–615 nm bandpass filter. Two‐color overlays were generated using the FIJI distribution of ImageJ. Particle coordinates data were collected and analyzed by Vernier Video Physics (version 3.0.5). Image processing was performed using ImageJ. Computational fluid dynamic modeling was done in COMSOL Multiphysics (version 5.4), using moving mesh method.

*Data Analysis*: See the Supporting Information for GO characterization data and methods.

*Droplet Rotation Analysis*: The raw data of the moving droplets were evaluated and processed using IBM SPSS software. The linear relationships of the droplets' angular displacement profiles were analyzed using a Pearson product‐moment correlation test.

## Conflict of Interest

The authors declare no conflict of interest.

## Supporting information

Supporting Information

Supplemental Video 1

Supplemental Video 2

Supplemental Video 3

Supplemental Video 4
