# Supplementary material for: Formation of Polarized, Functional Artificial Cells from Compartmentalized Droplet Networks and Nanomaterials, Using One‐Step, Dual‐Material 3D‐Printed Microfluidics
Source: Adv Sci (Weinh). 2019 Oct 24;7(1):1901719. doi: 10.1002/advs.201901719 (PMC6947711; doi:10.1002/advs.201901719)

Copyright WILEY-VCH Verlag GmbH & Co. KGaA, 69469 Weinheim, Germany, 2019.

ADVANCED SCIENCE
Open Access

Supporting Information

for *Adv. Sci.,* DOI: 10.1002/advs.201901719

Formation of Polarized, Functional Artificial Cells from Compartmentalized Droplet Networks and Nanomaterials, Using One-Step, Dual-Material 3D-Printed Microfluidics

Jin Li, Divesh Kamal Baxani, William David Jamieson, Wen Xu, Victoria Garcia Rocha, David Anthony Barrow, and Oliver Kieran Castell**

Copyright WILEY-VCH Verlag GmbH & Co. KGaA, 69469 Weinheim, Germany, 2019.

Supporting Information

Formation of polarised, functional artificial cells from compartmentalised droplet networks and nanomaterials, using one-step, dual-material 3D-printed microfluidics

Dr. J. Li[1,2], Dr, D. K. Baxani[1], Dr. W. D. Jamieson[1], W. Xu, Dr. V. G. Rocha[2], Prof. D. A. Barrow[2,]*, Dr. O. K. Castell[1,]*

[1]School of Pharmacy and Pharmaceutical Sciences, Redwood Building, King Edward VII Ave, Cardiff, CF10 3NB, United Kingdom
[2]School of Engineering, Queen's Buildings, 14-17 The Parade, Cardiff CF24 3AA, United Kingdom
E-mail: barrow@cardiff.ac.uk; castello@cardiff.ac.uk

Supplementary Method. GO Characterisation
Supplementary Discussion. CFD modelling
Table S1. Printing profiles of the 3D-printed microfluidic device within Cura.
Table S2. Additives for consistent emulsions formation in the 3D-printed microfluidic devices.
Table S3. CFD simulation results of the double emulsion droplet morphology in laminar flow.
Table S4. CFD simulation results of the morphology of double emulsion droplet with two types of inner droplets in laminar flow.
Table S5. The Pearson's correlations on the rotation of the highlighted hydrogel particles in Figure 5d.
Figure S1. Size distribution of the i_x*m_1 double emulsion droplets showing in Figure 3c.
Figure S2. Relative inner and middle droplet size under different input flow rates.
Figure S3. Characterization of the graphene oxide flakes.
Figure S4. An eDIB with a magnetic Janus shell and a droplet network, containing small (clear) and large (pink) droplets.
Figure S5. Images of eDIBs after three days of formation.
Video S1. Triple emulsion droplet generation in PLA+PVA 3D printed microfluidic devices.
Video S2. Simulation of the double emulsion droplet stabilisation within straight fluidic channel.
Video S3. eDIB generation in PLA+PVA 3D printed microfluidic devices
Video S4. Locomotion of the functionalised eDIBs with asymmetric magnetic shells in an aqueous environment.

Supplementary Method
GO Characterisation: X-ray photoelectron spectroscopy (XPS) analyses were performed on freeze dried GO using a K-Alpha spectrometer (ThermoFisher Scientific; East Grinstead, UK). XPS spectra were acquired using a microfocused monochromatic Al Kα X-ray source (hυ = 1486.6 eV). An X-ray spot of ~400 x 800 μm ellipse shape was used and three different areas were spotted. Core level C1s, CKLL, O1s, N1s, Mn1s, S2p, Na1s spectra were acquired using a pass energy of 200 eV and high regions at 40eV.
XPS Data Analysis: Casa XPS was used for data interpretation. Shirley or two point linear background subtractions were employed depending on background shape. Scofield cross-sections were used as relative sensitivity factors in the calculation of the atomic percentages (with RSF of C 1s = 1.000). Peaks were fitted using GL (30) lineshapes; a combination of a Gaussian (70%) and Lorentzian (30%). All XP spectra were charge corrected by referencing the fitted contribution of C-C graphitic like carbon in the C 1s signal to 284.6 eV. The atomic percentages were calculated from the peak areas in the acquired high resolution C 1s and O 1s photoelectron spectra using Scofield sensitivity factors.
FEGSEM Analysis: The morphology of the GO flakes drop casted on a Si/SiO2 wafer was analysed at 5 kV with field emission scanning electron microscopy on a LEO Gemini 1525 FEGSEM equipped with an energy dispersive spectroscopy microprobe (INCA Sight, Oxford Instruments, U.K.).

Supplementary Discussion:
Computational Fluid Dynamic (CFD) modelling of internal droplet dynamics and attainment of equilibrium:
Computational Fluid Dynamic (CFD) modelling was conducted with COMSOL Multiphysics software using a moving mesh method. The simulation results indicate that the water/oil/alginate double emulsion is stabilised under flow in the straight fluidic duct in our experimental conditions. This is due to the creation of high shear conditions within the oil droplet in close proximity to the oil-alginate interface generating a local opposing force repelling the internal aqueous droplet from the interfacial perimeter. In this first scenario (as shown in Figure 3a-iii), the internal aqueous droplet reaches its equilibrium position towards the front of the middle-phase oil droplet, with respect to the direction of flow (Table S3, Video S2). This morphology is consistent with our practical experiments, where it is observed that the internal aqueous droplets stabilise in position towards the front of the containing middle oil phase (e.g. see Figure 2-a1, inner aqueous droplet (pink), middle oil droplet (blue)). The internal aqueous phase is prevented from coalescing with the external aqueous-based alginate phase as a result of the competing forces of viscous stress and interfacial tension. The shear rate map of the modelled flows illustrates the hydrodynamic flow pattern within the double emulsion. Opposing, paired vortices within the middle oil phase, act to localise the internal droplet at its equilibrium position. Without this phenomenon and in the absence of effective surfactant stabilisation, coalescence of the aqueous phases could be expected to occur, resulting in internal droplet ejection from the oil-phase into the aqueous hydrogel continuous phase upon their contact. This effect and the resultant stabilisation of the multiphase emulsions is also dependent upon the linear flow velocity and droplet dimensions. In another scenario (as shown in Figure 3a-iv), multiple internal aqueous droplets are constrained at the waist of the middle oil droplets (Table S4). The joining of two single w/o emulsions at a Y-shaped junction forms a Janus oil phase, in which the internal droplets are constrained within their lateral domains of the encapsulating oil phase, rather than in the first scenario where the internal droplet is formed and migrates along the centreline of the fluidic channel. Within this second droplet morphology, the equilibrium

positions of the internal droplets become located at each side of the oil droplet midline (Video S2). We find that independent of internal aqueous droplet volumes tested, the droplets reach a symmetric lateral equilibrium position, approximately midway between the leading and trailing edge of the oil droplet, with respect to the direction of flow. Similarly, to the first simulation scenario, localised high shear regions at the flanks of the oil droplet limit the encroachment of the internal aqueous droplet to the oil droplet-alginate boundary. These fluid dynamic properties enable the maintenance of internal hemispherical patterning and encapsulated droplet hemispherical location within the oil droplet throughout the emulsion assembly process.

Table S1. Printing profiles of the 3D-printed microfluidic devices developed and used in this study. 3D projections reveal the use of three-dimensional channel geometries used in this study to define hemispherical patterning. Blue represents hydrophobic PLA printed material, yellow/white represent hydrophilic PVA, each defining preferential oil or aqueous phase channel wetting respectively. Left: channel outline, middle: complete microfluidic device manifold structure, right: mid-section slice of 3D manifold, revealing microfluidic channels within the mid-plane.

	inner duct structure (blue-hydrophobic, yellow-hydrophilic)	manifold structure (blue-PLA, yellow-PVA)	printing profiles, half-way (blue-PLA, white PVA)
1	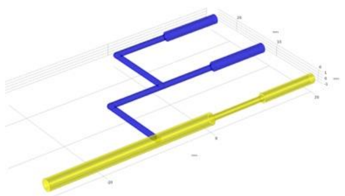	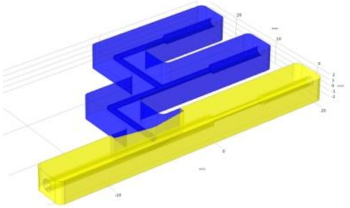	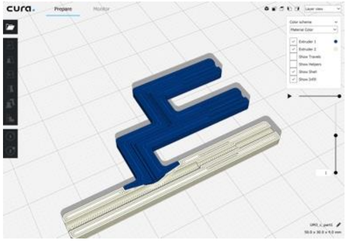
2	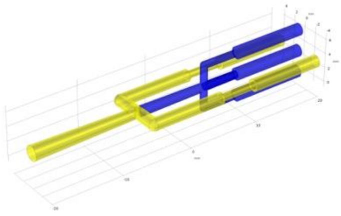	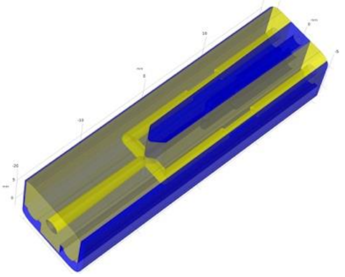	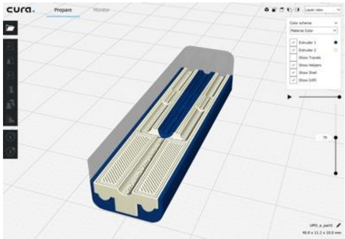
3	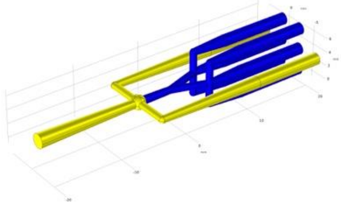	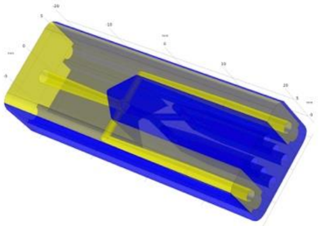	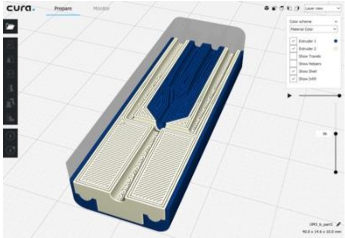
4	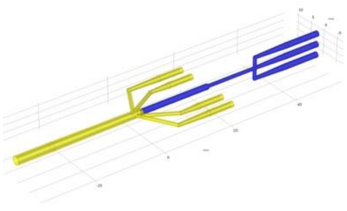	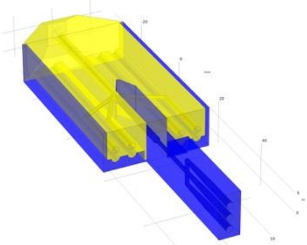	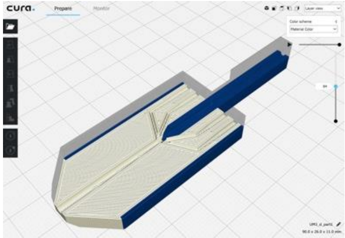

Table S2. Surfactants employed for consistent emulsion formation in the 3D-printed microfluidic devices.

Emulsions templates (inner phase/middle phase/outer phase)	Middle phase additives	Outer phase additives
Water/sunflower oil/water	n/a	0-2% v/v Tween20
Water/mineral oil/ 3% alginate	0-2% v/v Span80	0-6% v/v Tween20<br>or 0-2% w/v PVA powder
Water/hexadecane + silicone oil/ 3% alginate (double emulsion)	n/a	0-0.5% w/v PVA powder
Water/hexadecane + silicone oil/ 3% alginate (eDIBs)	5-8.3 mg/mL DPhPC	n/a

Table S3. CFD simulation results of the w/o/w double emulsion droplet morphology in laminar flow. The top schematic drawing indicated the droplet formation. The bottom table contains the initial status (t=0s) and equilibrium status (t=3s). The density maps indicate the phase distribution.

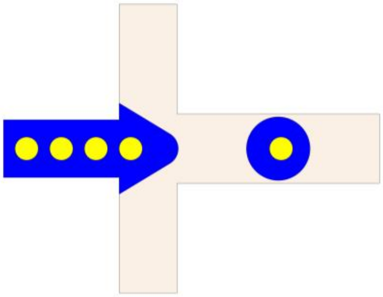

w/o/w	**inner droplets dia. = 0.4mm**	**inner droplets dia. = 0.8mm**
**t=0s, density map**	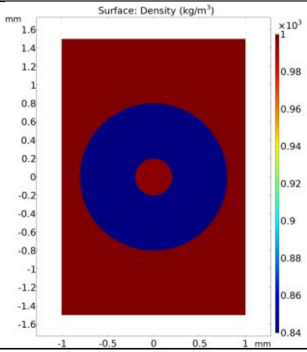	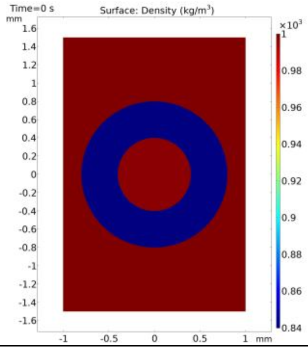
**t=3s, density map**	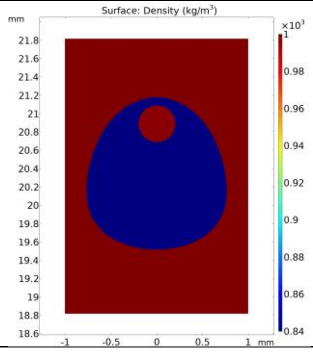	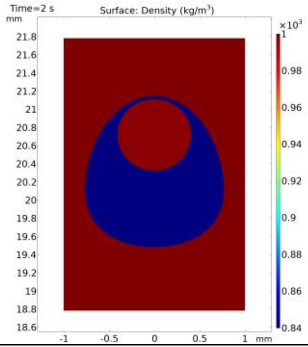
**t=3s, shear rate map**	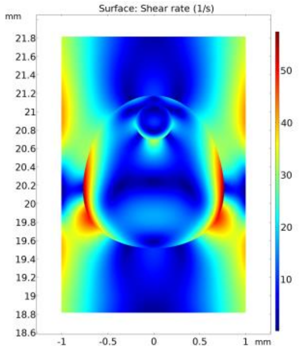	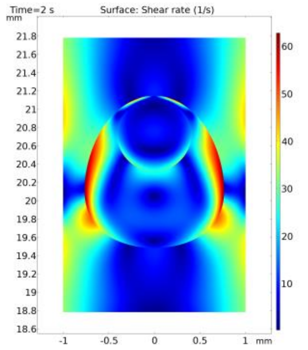

Table S4. CFD simulation results of the morphology of w/o/w double emulsion droplet with two types of inner droplets in laminar flow. The top schematic drawing indicated the droplet formation. The bottom table contains the initial status (t=0s) and equilibrium status (t=3s). The density maps indicate the phase distribution.

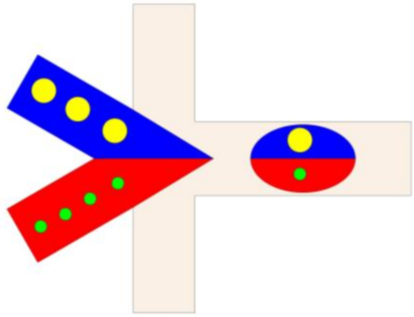

w/o/w	inner droplets:<br>left dia. = 0.1mm<br>right dia. = 0.2mm	inner droplets:<br>left dia. = 0. 2mm<br>right dia. = 0.2mm	inner droplets:<br>left dia. = 0.6mm<br>right dia. = 0.2mm
t=0s,<br>density<br>map	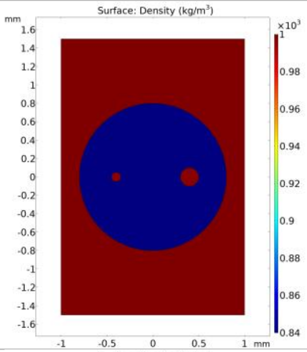	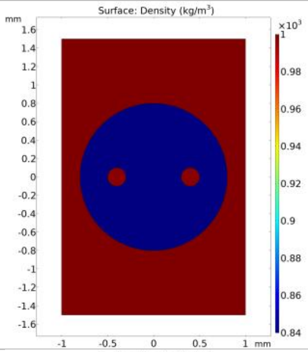	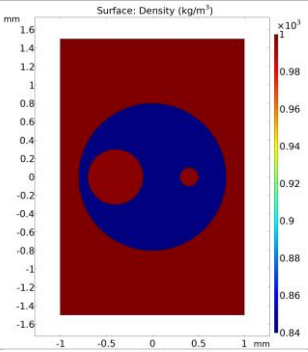
t=3s,<br>density<br>map	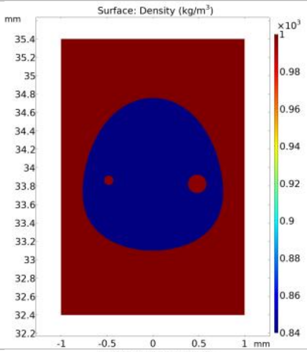	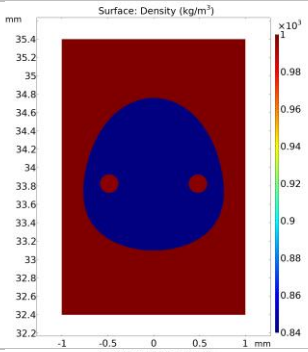	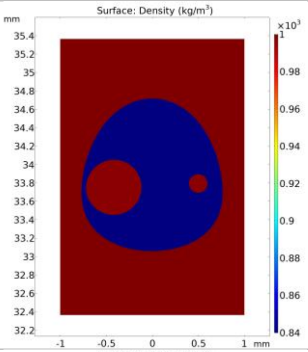
t=3s,<br>shear rate<br>map	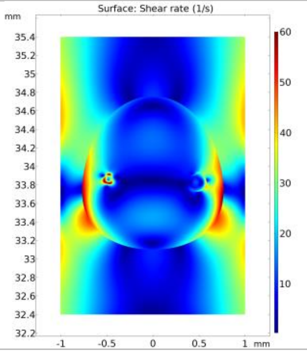	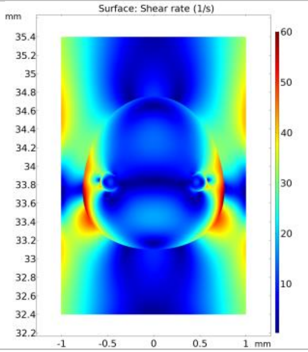	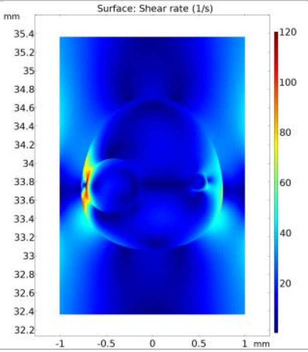

Table S5. The Pearson's correlations on the rotation of the highlighted hydrogel particles in Figure 5d. The angular displacements of particles orientation are highly correlated, which indicates that their rotations are synchronised and dominated by the external rotating magnetic field. The difference of the correlations may result from the temporal collision of particles (brown&blue). Stated colour corresponds to the coloured data points in Figure 5 d-ii

Correlations

		pink	green	brown	blue
pink	Pearson Correlation	1	.997**	.994**	.995**
	Sig. (2-tailed)		.000	.000	.000
	N	90	90	90	90
green	Pearson Correlation	.997**	1	.985**	.988**
	Sig. (2-tailed)	.000		.000	.000
	N	90	90	90	90
brown	Pearson Correlation	.994**	.985**	1	.997**
	Sig. (2-tailed)	.000	.000		.000
	N	90	90	90	90
blue	Pearson Correlation	.995**	.988**	.997**	1
	Sig. (2-tailed)	.000	.000	.000	
	N	90	90	90	90

**. Correlation is significant at the 0.01 level (2-tailed).

Figure S1. Size distribution of the i_x*m_1 double emulsion droplets showing in Figure 3c. n=20.

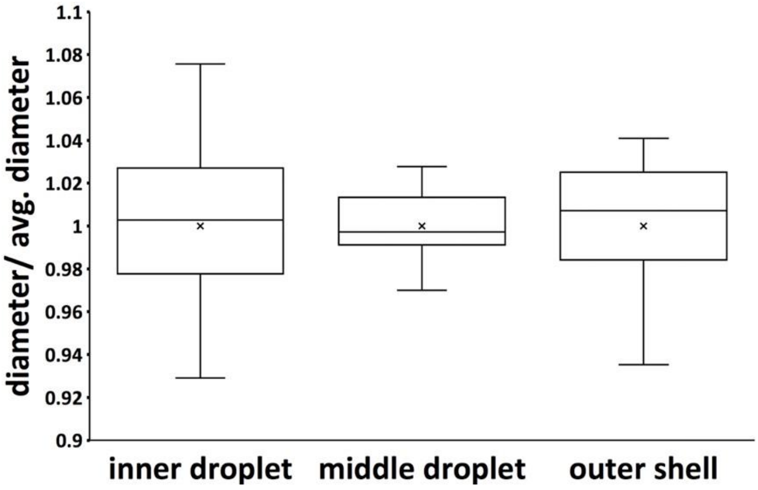

Figure S2. Graph of relative inner droplets and middle droplets size to the overall triple emulsion droplets under different input flow rate combinations using PLA+PVA 3d printed devices. These graphs are complementary with Figure 2b, describing the overall triple emulsion morphologies.

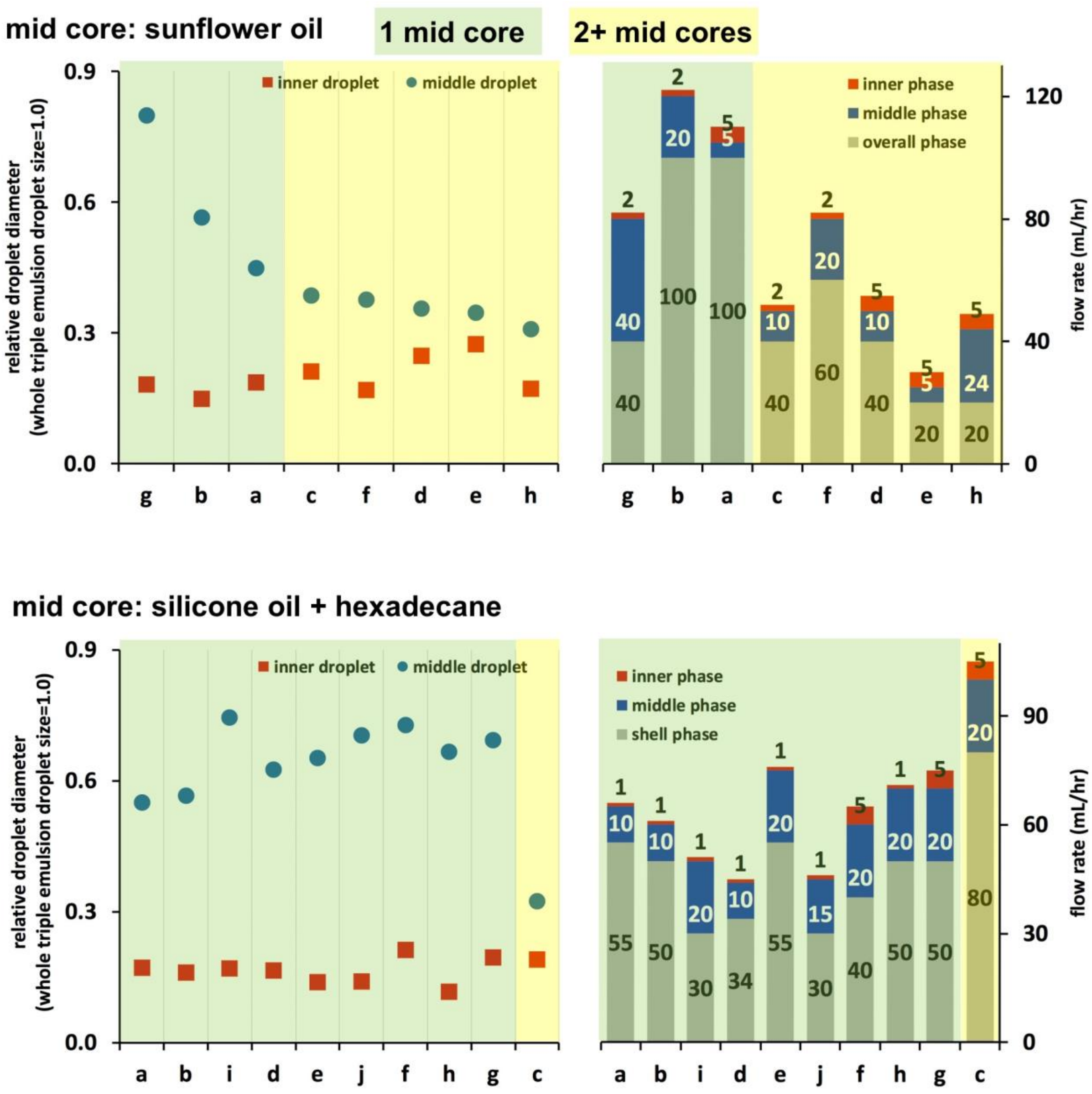

Figure S3. Characterization of the graphene oxide flakes. Optical microscope image of GO flakes on Si/SiO2 substrate obtained by differential interference contrast. (a) Field Emission Scanning Electron Microscopy of GO flakes. (b) Lateral size distribution for 100 GO flakes. (d) XPS of freeze dried GO. The XPS C1 signals were fitted to five components: C=C and C–C, C–O, C=O and COOR atomic percentage can be read in the graph.

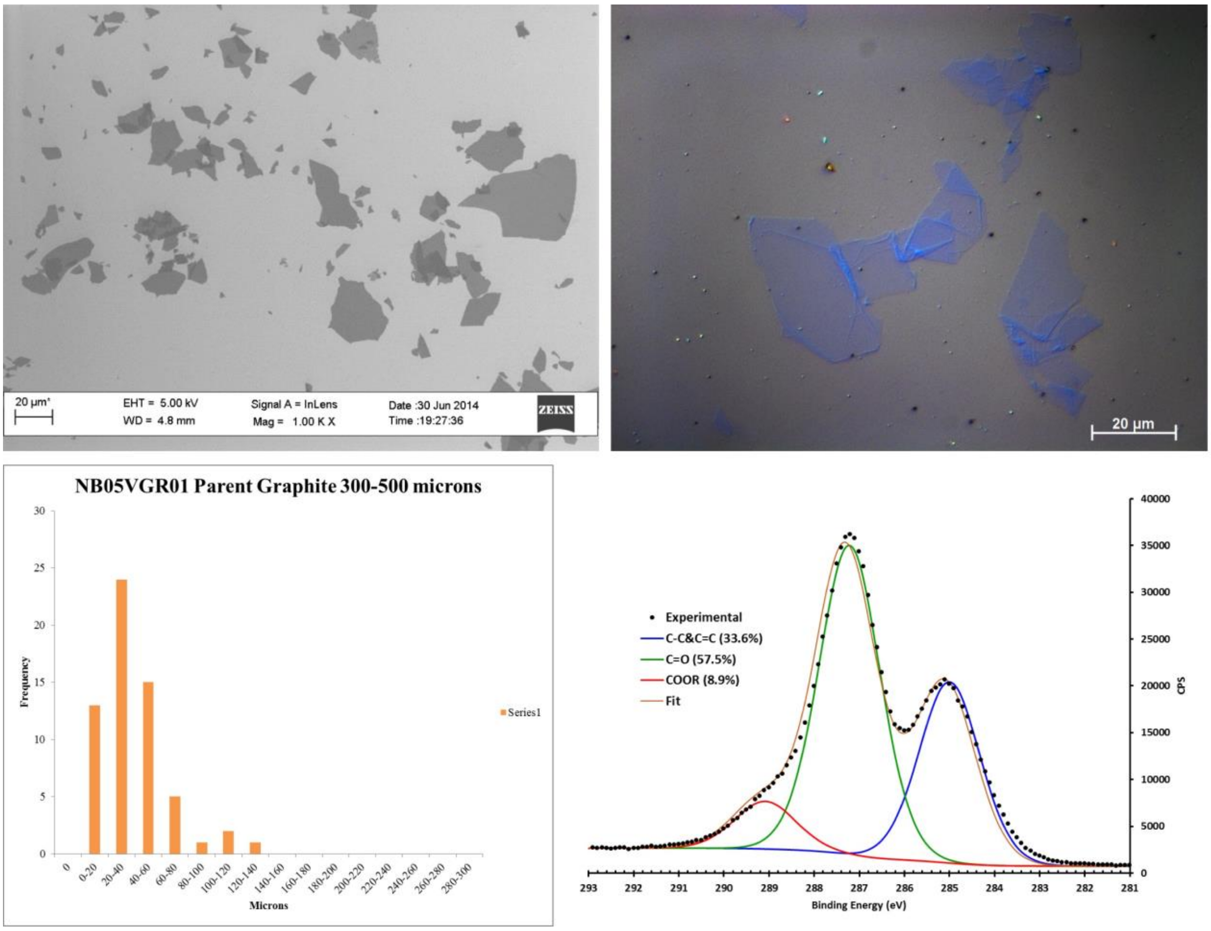

Figure S4. An eDIB with a magnetic Janus shell and a droplet network, containing small droplets (clear) on the left side and large (pink) droplets on the right side. Scale bar denotes 0.5 mm. The photo was taken from the bottom of the petri dish.

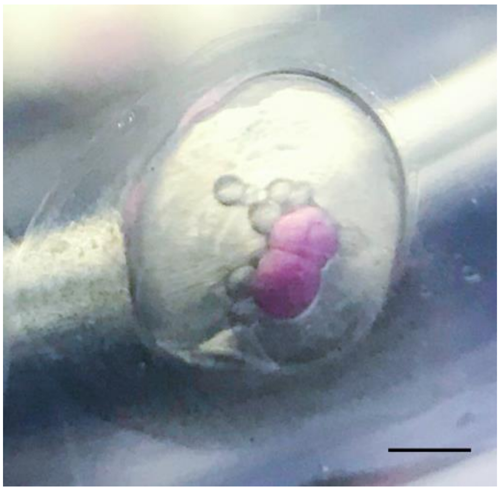

Figure S5. Images of eDIBs after three days of formation. Left column, fluorescent images, the red indicates the fluorescence from sulphrhodamine B within the internal aqueous droplets; right column, bright field images. Scale bars denote 1mm. In the first row (**a**), the dotted circle indicates that the top of the alginate shell was carefully severed for imaging the internal aqueous droplets (pink).

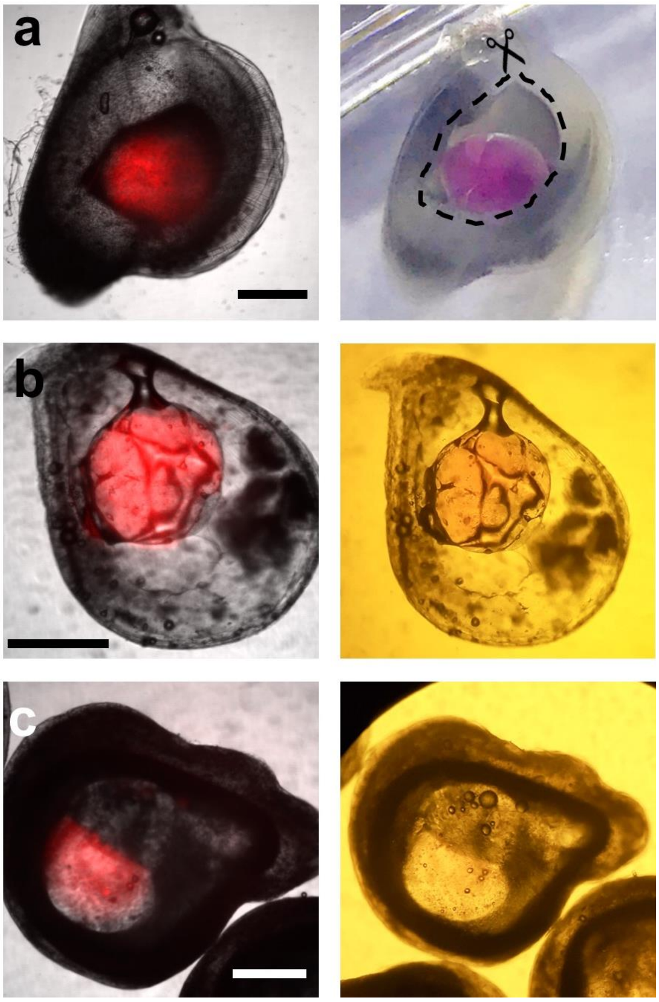

Supplement: Supplementary file 1 — Supporting Information [file ADVS-7-1901719-s001.pdf]
